# Epidemiology of the extent of recreational noise exposure and hearing protection use: cross-sectional survey in a nationally representative UK adult population sample

**DOI:** 10.1186/s12889-020-09602-8

**Published:** 2020-10-09

**Authors:** Christopher J. Armitage, Michael T. Loughran, Kevin J. Munro

**Affiliations:** 1 Manchester Centre for Health Psychology, School of Health Sciences, University of Manchester, Manchester, M13 9PL UK; 2 Manchester University NHS Foundation Trust, Manchester Academic Health Science Centre, Manchester, M13 9PL UK; 3 Manchester Centre for Audiology and Deafness, School of Health Sciences, University of Manchester, Manchester, M13 9PL UK

**Keywords:** Hearing loss, Hearing protection, Prevalence

## Abstract

**Background:**

Hearing loss is prevalent and disabling, yet little is known about the extent of recreational noise exposure and hearing protection use. The aim of the present research was to estimate the extent of recreational noise exposure and hearing protection use in a sample representative of the UK adult population.

**Methods:**

We conducted a cross-sectional survey of 10,401 UK adults who were representative of the population.

**Results:**

More than 7000 people (*n* = 7590, 73.0%) reported exposure to recreational noise excluding headphone and earphone use in the last 12 months. Just 158 people (2.1%) reported wearing hearing protection for every noisy recreational activity. Age (younger people) and beliefs of a behavioral (as opposed to genetic) cause of hearing loss were predictive of both higher recreational noise exposure and greater hearing protection use. Men were more exposed to recreational noise but women were less likely to use hearing protection.

**Conclusions:**

For the first time, the present research quantifies the recreational noise exposure and low levels of hearing protection use in a representative sample of the UK population. The biggest public health gains are likely to be achieved through interventions targeted at younger people and in explaining behavioral (as opposed to genetic) causes of hearing loss.

## Background

Hearing loss is ranked 4th globally in years lived with a disability, with older age and noise exposure being the biggest risk factors [1]. Approximately one billion teenagers and young adults (12–35 years) are at risk of noise-induced hearing loss due to hazardous recreational listening behaviors [1]. These behaviors include attendance at live music venues (nightclubs, festivals, concerts and bars), practising/producing music, DIY, engine noise and sports related noise [2–6]. In many instances, the risks of recreational noise exposure can be reduced through the use of hearing protection devices (earplugs and earmuffs).

Numerous studies have tried to estimate recreational noise exposure and hearing protection uptake (e.g., use of earplugs and/or earmuffs), but have typically been limited to convenience [7–13] or regional samples [14–17] resulting in estimates of exposure and protection use ranging between 9 and 51%, and 2–61%, respectively. We were able to identify one study [18] that attempted to assess the prevalence of recreational noise exposure and use of hearing protection in a nationally representative sample of 18–35 year olds but their final sample included 76% women and was therefore not nationally representative, even ignoring the limited age range. Thus, little is known about the extent of recreational noise exposure and hearing protection use among the general population, beyond samples of adolescents and University students.

Without knowledge of the extent to which the general public are exposed to recreational noise and what is the prevalence of hearing protection device use, it will be difficult to develop interventions to reduce the health costs that might arise in the future nor what interventions might promote use of hearing protection. The aims of the present research are, for the first time in a representative sample of the UK adult population, to: (a) assess the extent of recreational noise exposure, and (b) assess hearing protection use.

Once the frequency of recreational noise exposure and prevalence of hearing protection use is established, the question then arises as to what are the correlates of exposure and use that will shed light on the kinds of populations and constructs that may be targeted/changed in future interventions. We therefore additionally examined potential correlates of recreational noise exposure and hearing protection use, in addition to sociodemographic variables and clinical characteristics (e.g., current hearing loss), we also considered beliefs about whether hearing loss is caused by genetics or health behaviors [19] to see whether public perceptions may need to be changed as well.

## Methods

### Design

The study design was cross-sectional and administered via an online survey.

### Participants

A sample of adults designed to be representative of the UK adult (18+ years) population was invited by YouGov, a market research company, to take part in an online questionnaire in March 2019. YouGov has a panel of over one million potential respondents from whom they successfully recruited a sample of 10,401 UK residents, based on a 90.6% completion rate. Participants were incentivized in accordance with YouGov’s points system, whereby respondents accumulate points for taking part in online surveys and the points can be traded for cash and gift cards. The data were sent securely to the research team for analysis. Ethical approval was obtained from a University of Manchester ethics committee (ref: 2019–5769-9246) and participants gave written informed consent by clicking on a series of boxes at the beginning of the online survey.

### Procedure

A questionnaire, designed specifically for the purposes of the present study, was embedded in a wider online anonymous survey. We asked questions about participants’ sociodemographic and clinical characteristics, recreational noise exposure, hearing protection use and beliefs about the causes of hearing loss. These questions are described briefly in the section below and are presented in the Additional file 1. They include a mixture of standard items (e.g., sociodemographic characteristics) and standard items that were adapted for the present purposes (e.g., causal beliefs).

#### Sociodemographic and clinical characteristics

Sociodemographic measures of gender, ethnicity and socioeconomic status were taken using standard UK Office for National Statistics (2016) measures. Consistent with the ethnic profile of the UK, ethnicity was divided into White versus Black, Asian or Minority Ethnic. We gathered data on twelve occupational categories (see Additional file 1), but found no differences and so reported as manual versus non-manual. Participants were asked two questions derived from Benova et al. [20] to assess personal/familial experiences with hearing loss: “Do you have any difficulty with your hearing?” and “Do any of your family members currently have hearing loss?” to which participants were asked to respond “no” or “yes.”

#### Causal beliefs

Participants’ causal beliefs were assessed using measures adapted from Nguyen et al. [19] and were asked to respond to items regarding their beliefs about the roles of genetics and behavior in developing hearing loss. With respect to *genetic causal beliefs*, participants were asked, “How much do you think genetics, that is characteristics passed from one generation to the next, determine whether or not a person will develop hearing loss?”; *behavioral causal beliefs* were assessed with the question, “How much do you think health behaviors like diet, exercise and smoking determine whether or not a person will develop hearing loss?”. Responses to both questions were given on identical four-point scales: *not at all*, *a little*, *somewhat*, and *a lot*.

#### Extent of recreational noise exposure

Participants were presented with a definition of “noisy leisure activities”: A “noisy leisure activity” is where you need to raise your voice to be heard when at arms-length from someone who has normal hearing. Do not include times when you are using headphones/earphones. Noisy activities can cover a wide range of settings such as: live music (e.g. concerts, festivals), nightlife (e.g. clubs, bars, pubs), making music (e.g. in a band, home producer), DIY (e.g. power tools, powered gardening tools), engine noise (e.g. motorbikes, motorboats, motorsports), sports related noise (e.g. watching rugby or football matches live, firearms and fireworks) or cinema.” Following this, participants were asked, “How often did you take part in noisy leisure activities during the last 12 months?” [7, 15] to which they responded on an eight-point scale: “Almost every day,” “5 or 6 times a week,” “3 or 4 days a week,” “Once or twice a week,” “Once or twice a month,” “Once every couple of months,” “Once or twice a year,” or “Not at all in last 12 months.” Responses were coded such that higher values indicate greater exposure to noise during leisure activities.

#### Hearing protection use

Use of hearing protection during exposure to noisy leisure activities was measured using the item, “How often, if at all, do you use earplugs and/or earmuffs to protect your hearing when you take part in noisy leisure activities?” to which participants responded on an eleven-point scale from 0 to 100% that had 10% intervals, which was adapted for questionnaire use from Lutman et al. [21].

### Analyses

Data were weighted to ensure analyses properly reflected the UK population. Descriptive statistics were used to characterize the population. Linear multiple regression was used to predict recreational noise exposure and logistic regression was used to identify predictors of hearing protection use.

## Results

### Participant characteristics

Consistent with the sampling frame, the sample was broadly representative of the UK population (Table 1). Most participants were white (93.9%) and half were women (51.0%) and roughly evenly split between people in non-manual (51.6%) and manual occupations (48.4%). Mean age was 47.41 years (*SD* = 1.48; 18–93 years). Most people (75.9%) did not report a hearing loss and did not have people in their family with hearing loss (67.4%). Approximately half (49.8%) of the sample believed that hearing loss was caused by genetics whereas just 16.8% thought that hearing loss had a behavioral cause.

**Table 1:** Characteristics of the Sample

Variable	%	*M*	*SD*
Gender			
Men, *n* = 5007	48.1	–	–
Women, *n* = 5305	51.0	–	–
Age	–	47.41 years	1.48 years
18–35 years	31.3		
36–65 years	49.4		
66–93 years	19.3		
Socioeconomic Status			
Non-manual, *n* = 5367	51.6	–	–
Manual, *n* = 5034	48.4	–	–
Ethnicity			
White, *n* = 9764	93.9	–	–
Black, Asian and Minority Ethnic/Prefer not to say, *n* = 637	6.1	–	–
Personal Hearing Difficulty			
Yes, *n* = 2510	24.1		
No, *n* = 7891	75.9		
Family Hearing Difficulty			
Yes, *n* = 3390	32.6		
No, *n* = 7011	67.4		
Genetic Causal Beliefs (1 = *not at all*; 4 = *a lot*)		2.43	0.96
Behavioral Causal Beliefs (1 = *not at all*; 4 = *a lot*)		1.70	0.83
Extent of Recreational Noise Exposure in the Last 12 Months (*Median* = “Once every couple of months”)		2.90	1.70
Hearing Protection Use (*Median* = 20% of occasions exposed)		1.90	2.25

*Note*. Values that do not add up to *N* = 10,401 indicate that participants ‘preferred not to say’

### Recreational noise exposure

**Fig. 1:**
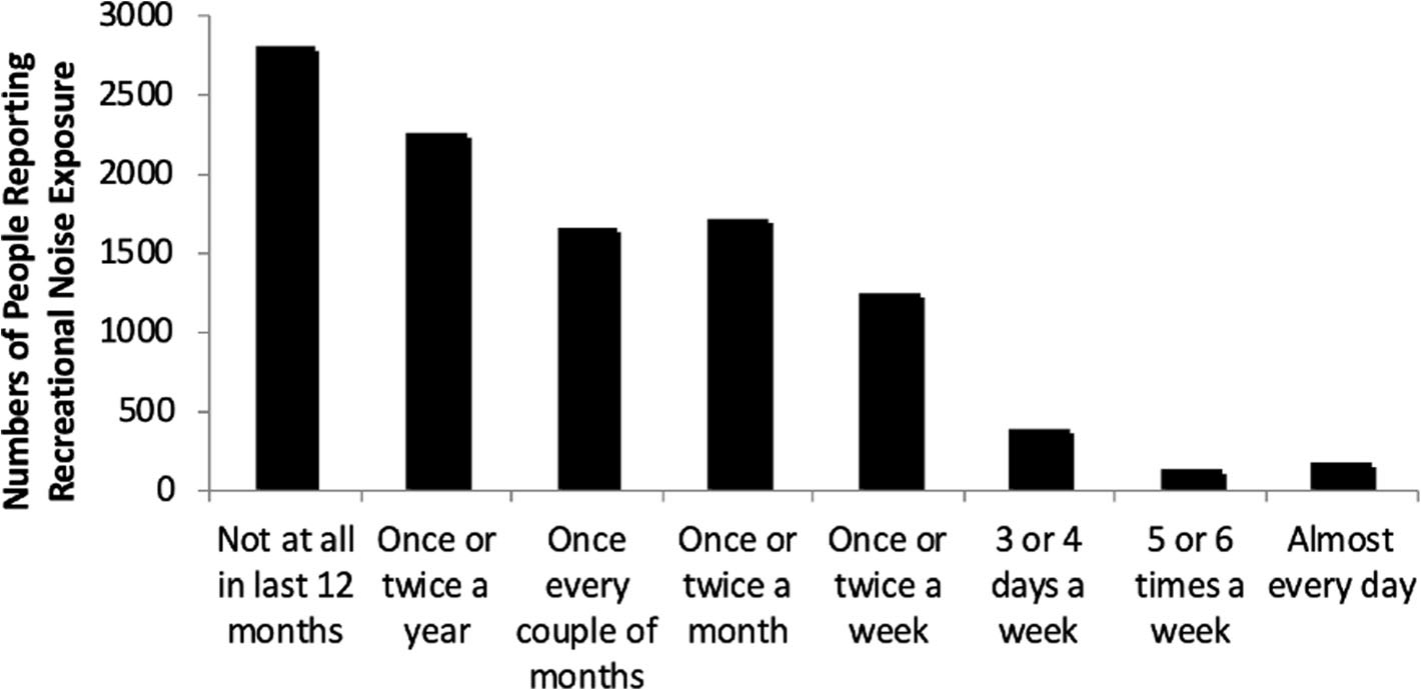
Frequency of Recreational Noise Exposure

The majority of participants were exposed to recreational noise on only an occasional basis: 64.7% (*n* = 6732) reported experiencing recreational noise less than once a month (Fig. 1). Nevertheless a significant minority (18.8%, *n* = 1951) reported exposure to excessive recreational noise on at least a weekly basis. Multiple regression (Table 2) showed that men, younger people, people with a Black, Asian or Minority Ethnic background and those with personal experience of hearing loss were more likely to have been exposed to excessive recreational noise in the previous 12 months. Exposure was also associated with weaker beliefs in a genetic cause of hearing loss, stronger beliefs in a behavioral cause of hearing loss and greater use of hearing protection.

**Table 2:** Predictors of Recreational Noise Exposure

Independent Variables	*B*	*SE*	95% CI
Gender (men = 1; women = 2)	−.31	.03	−.38, −.25*
Age	−.01	.01	−.02, −.01*
Socioeconomic Status (1 = non-manual; 2 = manual)	.04	.03	−.03, .10
Ethnicity (1 = White; 2 = Black, Asian and Minority Ethnic/Prefer not to say)	.28	.07	.14, .42*
Personal Hearing Difficulty	.29	.04	.21, .37*
Family Hearing Difficulty	.10	.04	.03, .17*
Genetic Causal Beliefs	−.04	.02	−.08, −.01*
Behavioral Causal Beliefs	.08	.02	.04, .12*
Hearing Protection Use	.33	.04	.25, .41*

*Note.* Greater exposure is associated with being a man, being younger, being from a Black, Asian and Minority Ethnic Background, experiencing personal hearing difficulty, having a family history of hearing difficulty, weaker genetic causal beliefs, stronger behavioral causal beliefs and greater hearing protection use

*p < .05

### Hearing protection use

**Fig. 2:**
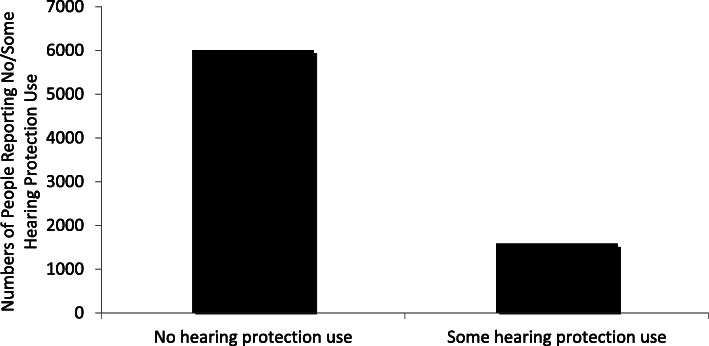
Use of Hearing Protection Among People Exposed to Recreational Noise

More than 7000 people (*n* = 7590, 73.0%) reported some exposure to recreational noise in the last 12 months. Of those participants reporting some recreational noise exposure, *n* = 6007 (79.2%) reported zero use of hearing protection during noisy recreational activities in the last 12 months (Fig. 2); just 158 people (2.1%) reported wearing hearing protection for every noisy recreational activity. The subsequent analyses focus on the 7590 participants who reported some exposure and hearing protection use was dichotomized into “no use” versus “some use” in the last 12 months.

Univariate and multivariate logistic regression was used to identify correlates of hearing protection use (Table 3). Four significant predictors emerged from both the univariate and the multivariate analyses. Men, younger people, stronger beliefs in a behavioral cause of hearing loss and greater exposure were all associated with greater hearing protection use.

**Table 3:** Predictors of Hearing Protection Use

Independent Variables	*n* (%) Hearing Protection Users	OR (95% CI)	Adjusted OR (95% CI)
Gender		0.57 (0.51, 0.64)*	0.60 (0.54, 0.68)*
Men	938 (25.4)		
Women	623 (16.2)		
Age		0.99 (0.98, 0.99)	0.99 (0.99, 0.99)*
Socioeconomic Status		0.97 (0.86, 1.08)	1.00 (0.89, 1.12)
Non-manual	899 (21.1)		
Manual	683 (20.5)		
Ethnicity		1.49 (1.20, 1.84)	1.24 (0.99, 1.55)
White	1457 (20.4)		
Black, Asian and Minority Ethnic/Prefer not to say	126 (27.7)		
Personal Hearing Difficulty		0.99 (0.87, 1.13)	1.06 (0.92, 1.22)
Yes	383 (20.8)		
No	1199 (20.9)		
Family Hearing Difficulty		0.99 (0.88, 1.11)	1.09 (0.96, 1.23)
Yes	529 (20.7)		
No	1053 (20.9)		
Genetic Causal Beliefs		1.06 (1.00, 1.12)	0.98 (0.92, 1.04)
Behavioral Causal Beliefs		1.34 (1.25, 1.43)*	1.27 (1.19, 1.37)*
Extent of Exposure		1.22 (1.17, 1.26)*	1.16 (1.12, 1.21)*

*Note*. Adjusted analyses include all predictors in the model. Greater hearing protection use is associated with being a man, being younger, having stronger behavioral causal beliefs and having greater exposure to recreational noise

*p < .05

## Discussion

This study is the first of its kind to examine the extent of recreational noise exposure in the UK and associated hearing protection use. The key findings are that 73.0% of people reported exposure to recreational noise in the last 12 months and that hearing protection use is very low with 2.1% reporting wearing hearing protection for every noisy recreational activity. Experience of hearing loss is associated with greater exposure to recreational noise but not greater use of hearing protection. The following discussion considers the practical and policy implications of this work.

The present study highlights a substantial gap, among a representative sample of UK adults, between the extent of exposure to recreational noise and use of hearing protection that is similar to the rates found in students in the US [7] and Belgium [15]. The implication is that there is still much work to be done in raising awareness of recreational noise exposure and in promoting hearing protection use.

The present study sheds some light on the kinds of populations that may need to be targeted and the kinds of beliefs that might need to be changed to prevent recreational noise exposure and/or promote increase hearing protection use. The key findings were that: (a) interventions to reduce exposure might best be targeted at men and younger people, and that more needs to be done in terms of explaining that there is a behavioral underpinning to hearing loss; and (b) interventions to increase hearing protection use might best be targeted at women and older people with challenges made to the belief that hearing loss has a genetic cause.

Ensuring that people are aware of the behavioral underpinnings to hearing loss is something that could be implemented at a population level, rather than targeting specific groups and it is notable that exposure was associated with greater reported hearing loss. The implication is that perhaps exposure drives the belief that hearing loss has a behavioral as opposed to genetic cause of hearing loss, which chimes with research conducted in the US showing that the presence of hearing symptoms contributed to greater negative attitudes towards noise [7]. The implication is that early interventions to ensure that people are aware of behavioral causes of hearing loss prior to exposure are required.

### Strengths and limitations

Although the present research takes the literature on the prevalence of recreational noise exposure and health protection use forward in some important respects, it is important to note some potential limitations. First, although the sample was large and representative, the cross-sectional design of the study means that causality cannot be inferred. Second, the nature of the research meant that exposure and use of hearing protection were self-reported, rather than assessed objectively. It would be valuable in future research to harness the power of new technology to gauge population exposure to recreational noise and use of hearing protection unobtrusively with a greater level of accuracy. Third, it is worth highlighting that, as a function of our interest in both recreational noise exposure and hearing protection use, our definition of noisy recreational activities excludes headphone and earphone use. This means that our estimate of exposure is likely to be lower than what has traditionally been considered to be total recreational noise exposure (i.e., including headphone and earphone use).

## Conclusions

In sum, a significant proportion of the UK population are exposed annually to recreational noise, yet few take protective action. Younger people and men might be appropriate target groups to reduce exposure; whereas older people and women might be targeted with interventions to increase hearing protection use. However, a population-level approach to improving hearing health might usefully focus on ensuring that people have greater awareness of the behavioral causes of hearing loss.

## Supplementary information

**Additional file 1.**

## Data Availability

The dataset used during the current study are available from the corresponding author on reasonable request.
