# Supplementary material for: Epidemiology of the extent of recreational noise exposure and hearing protection use: cross-sectional survey in a nationally representative UK adult population sample
Source: BMC Public Health. 2020 Oct 9;20:1529. doi: 10.1186/s12889-020-09602-8 (PMC7547427; doi:10.1186/s12889-020-09602-8)
Supplement: Supplementary file 1 — Additional file 1. [file 12889_2020_9602_MOESM1_ESM.doc]

Questionnaire

1. Gender

☐Female

☐Male

☐Female/Male does not describe me, I prefer…________________

1. Age (in years)

(Drop down menu)

1. How would you describe your ethnicity?

☐ White British

☐ White Irish

☐ Any other white background

☐ Mixed – white and black Caribbean

☐ Mixed – white and black African

☐ Mixed – white and Asian

☐ Any other mixed background

☐ Asian or Asian British - Indian

☐ Asian or Asian British – Pakistani

☐ Asian or Asian British – Bangladeshi

☐ Any other Asian or Asian British background

☐ Black or black British – Caribbean

☐ Black or black British – African

☐ Any other black or black British background

☐ Chinese

☐ Other (please specify) ________________________

1. What is your occupation?

☐ Management

☐ Technical / IT

☐ Professional

☐ Administrative

☐ Sales / Support

☐ Production / Manufacturing

☐ Healthcare & Social Assistance

☐ Other employment

☐ Unemployed

☐ Student

☐ Retired

☐ Other

1. Do you have any difficulty with your hearing?

☐Yes

☐No

1. Do any of your family members currently have hearing loss?

☐Yes

☐No

If yes, how many? **_______ family members**

1. How much do you think health behaviours like diet, exercise and smoking determine whether or not a person will develop hearing loss?

| Not at all | A little | Somewhat | A lot |
| --- | --- | --- | --- |

1. How much do you think genetics, that is characteristics passed from one generation to the next, determine whether or not a person will develop hearing loss?

| Not at all | A little | Somewhat | A lot |
| --- | --- | --- | --- |

Now we want you to think about any noisy leisure activities that you might take part in. A “noisy leisure activity” is where you need to raise your voice to be heard when at arms-length from someone who has normal hearing. Do not include times when you are using headphones/earphones. Noisy activities can cover a wide range of settings such as: live music (e.g. concerts, festivals), nightlife (e.g. clubs, bars, pubs), making music (e.g. in a band, home producer), DIY (e.g. power tools, powered gardening tools), engine noise (e.g. motorbikes, motorboats, motorsports), sports related noise (e.g. watching rugby or football matches live, firearms and fireworks) or cinema.

1. How often did you take part in noisy leisure activities during the last 12 months?

☐ Almost every day

☐ 5 or 6 times a week

☐ 3 or 4 days a week

☐ Once or twice a week

☐ Once or twice a month

☐ Once every couple of months

☐ Once or twice a year

☐ Not at all in last 12 months

1. How often, if at all, do you use earplugs and/or earmuffs to protect your hearing when you take part in noisy leisure activities?

Percentage of noisy leisure activities for which hearing protection is used:

*No activities* ► **0% 10% 20% 30% 40% 50% 60% 70% 80% 90% 100%** ◄ *All activities*
